# Repurposing of antibiotics for clinical management of COVID-19: a narrative review

**DOI:** 10.1186/s12941-021-00444-9

**Published:** 2021-05-21

**Authors:** Abdourahamane Yacouba, Ahmed Olowo-okere, Ismaeel Yunusa

**Affiliations:** 1 Faculté des Sciences de la Santé, Université Abdou Moumouni, P.M.B. 10896, Niamey, Niger; 2 Faculty of Pharmaceutical Sciences, Usmanu Danfodiyo University, P.M.B. 2346, Sokoto, Nigeria; 3 Department of Clinical Pharmacy and Outcomes Sciences, University of South Carolina College of Pharmacy, Columbia, SC USA

**Keywords:** COVID-19, SARS-CoV-2, Drug repurposing, Antibiotics

## Abstract

**Background:**

Drug repurposing otherwise known as drug repositioning or drug re-profiling is a time-tested approach in drug discovery through which new medical uses are being established for already known drugs. Antibiotics are among the pharmacological agents being investigated for potential anti-SARS-COV-2 activities. The antibiotics are used either to resolve bacterial infections co-existing with COVID-19 infections or exploitation of their potential antiviral activities. Herein, we aimed to review the various antibiotics that have been repositioned for the management of COVID-19.

**Methods:**

This literature review was conducted from a methodical search on PubMed and Web of Science regarding antibiotics used in patients with COVID-19 up to July 5, 2020.

**Results:**

Macrolide and specifically azithromycin is the most common antibiotic used in the clinical management of COVID-19. The other antibiotics used in COVID-19 includes teicoplanin, clarithromycin, doxycycline, tetracyclines, levofloxacin, moxifloxacin, ciprofloxacin, and cefuroxime. In patients with COVID-19, antibiotics are used for their immune-modulating, anti-inflammatory, and antiviral properties. The precise antiviral mechanism of most of these antibiotics has not been determined. Moreover, the use of some of these antibiotics against SARS-CoV-2 infection remains highly controversial and not widely accepted.

**Conclusion:**

The heavy use of antibiotics during the COVID-19 pandemic would likely worsen antibiotic resistance crisis. Consequently, antibiotic stewardship should be strengthened in order to prevent the impacts of COVID-19 on the antibiotic resistance crisis.

## Introduction

In December 2019, a pneumonia like disease of unknown cause emerged in Wuhan, an emerging business hub located in the Hubei province of China [1]. The disease was caused by a highly transmissible, hitherto undescribed beta-coronavirus, the *SARS-coronavirus-2* (SARS-CoV-2) [2, 3]. The disease rapidly spread globally prompting the World Health Organisation (WHO) to declare it a global pandemic in March, 2020 [4]. As of 24th November 2020, 59,175,309 laboratory-confirmed COVID-19 cases were reported worldwide, with 1,396,403 deaths [5].

The rising biological, clinical, and socio-economic impacts of this COVID-19 diseases underscore the urgent need for effective resolution of this crisis [6, 7]. Currently, there is no specific vaccine or an approved antiviral for its effective treatment, several strategies are however being explored [3]. Drug repurposing offers a quick and cost-effective strategy to achieve this [8]. Drug repurposing otherwise known as drug repositioning or drug re-profiling is a time-tested approach in drug discovery through which new medical uses are being established for already known drugs, including approved, discontinued, shelved and experimental drugs [8]. This approach offers considerable advantage over the search for novel molecules. The advantages of drug repurposing have been summarised in a published review article on drug repurposing [8]. This approach has been successful used to brought back several drugs to the market [9]. Zidovudine for example, a well-known antiviral drug active against human immunodeficiency virus (HIV) has been shown to demonstrate in-vitro activity against colistin-resistant and carbapenem-resistant isolates [10]. Similarly, some anti-cancer drugs have been successfully repurposed for treatment of resistant bacterial infections [11]. Other successful examples abound in the literature.

Currently, various pharmacological agents are being investigated for potential use in the clinical management of coronavirus diseases [12–15]. The inclusion of antibiotics in the clinical management of COVID-19 is aimed at achieving either the resolution of any bacterial infections co-existing with the COVID-19 infections or exploitation of its potential antiviral activities. Bacterial co-infection is common feature in Covid-19 diseases [16]. As much as 94.2% patients with confirmed cases of COVID-19 diseases in China have been found to be co-infected with one or more other pathogens [17]. In another study, 51.35% of paediatric patients with COVID-19 diseases were also co-infected with other pathogens [18]. The prominent use of antibiotics in the clinical management of COVID-19 diseases is therefore not out of place. In this article, we aimed to review the various antibiotics that have been repositioned for clinical management of COVID-19 diseases. This review focuses on the current state of knowledge regarding the repurposing of antibiotics in terms of their modes of action, antiviral efficacy, and the advances to-date in their development as antiviral agents for clinical use.

## Methods

### Literature search strategy

A methodical search of PubMed and Web of Science was conducted to identify articles published up till July 5, 2020 that involved studies on repurposing of antibiotics for clinical management of COVID-19 diseases.

The following ‘Medical Subject Headings’ (MeSH) terms and text words were used to search articles in PubMed: (Drug Repurposing or Drug Re-profiling or Drug re-positioning) AND (Antibiotics.mp.) OR (Anti-Bacterial Agents) OR (Antimicrobial agents.mp. or Anti-Infective Agents) AND (Coronavirus disease) OR (COVID 19.mp.) OR (SARS-coronavirus-2 diseases.mp.) The following keywords were used to search articles in Web of Science: (“Drug Repurposing” or “Drug Re-profiling” or “Drug re-positioning”) AND (“Antibiotics” OR “Anti-Bacterial Agents”) AND (“COVID-19” OR “corona virus disease” OR “SARS-coronavirus disease”). In addition, Google Scholar was also searched for articles with the appropriate keywords. References of identified were also searched.

## Results

**Fig. 1:**
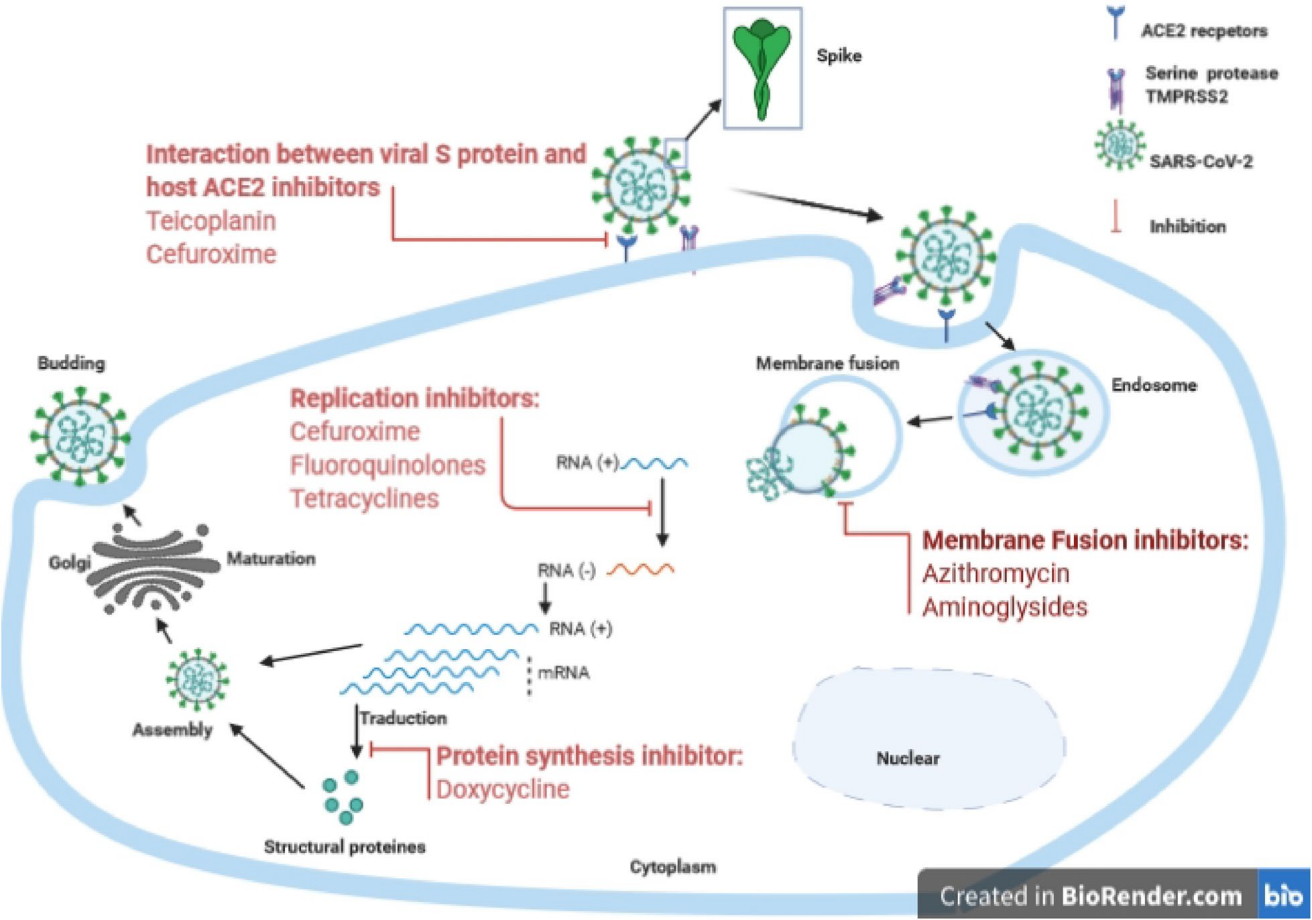
Scheme of potential targets of repurposed antibiotics against SARS-CoV-2

Collected data related to the use of antibiotics in COVID-19 (up to July 5, 2020) are summarised in Table 1. Figure 1 shows the scheme of potential targets of repurposed antibiotics against SARS-CoV-2.

**Table 1:** Collected data related to the use of antibiotics in COVID-19 (up to July 5, 2020)

Authors name + reference	Antibiotics	Types of study	Potential viral targets and/or other properties	IC50 inhibition or posology
Pani et al. [82]	Azithromycin	Review	Anti-inflammatory and immunomodulatory effects	Not indicated
Choudhary et al. [83]	Azithromycin	Review	Membrane fusion inhibition	Not indicated
Gautret et al. [26]	Azithromycin	Non-randomized clinical trial	Membrane fusion inhibition	500 mg on the first day then 250 mg/day for 5 more days
Andreani et al. [32]	Azithromycin	In vitro	Membrane fusion inhibition	10 and 5 μM
Touret et al. [84]	Azithromycin, levofloxacin	In vitro	Membrane fusion inhibition and replication inhibition	Not indicated
Ceccarelli et al. [42]	Teicoplanin	Letter to the editor	Interaction between viral spike protein and ACE2 receptors inhibition	6 mg/kg every 24 h
Baron et al. [41]	Teicoplanin	Editorial	Interaction between viral spike protein and ACE2 receptors inhibition	Not indicated
Zhang et al. [43]	Teicoplanin	Original article	Interaction between viral spike protein and ACE2 receptors inhibition	1.66 µΜ
Sathyamoorthy et al. [85]	Teicoplanin	Letter to the editor	Interaction between viral spike protein and ACE2 receptors inhibition	Not indicated
He and Garmire [86]	COL-3 (a chemically modified tetracycline)	Computational study	Interaction between viral spike protein and ACE2 receptors inhibition	Not indicated
Sodhi and Etminan [87]	Tetracyclines	Letter to the editor	Zinc‐chelating and anti-inflammatory effects	Not indicated
Wang [58]	Eravacycline, streptomycin	Computational study	Replication inhibition	Not indicated
Conforti et al. [88]	Doxycycline	Letter to the editor	Anti-inflammatory effect	Not indicated
Farouk and Salman [89]	Doxycycline	Letter to the editor	Anti-inflammatory effect	Not indicated
Malek et al. [90]	Doxycycline	Editorial	Anti-inflammatory effect	Not indicated
Szolnoky [91]	Doxycycline	Letter to the editor	Anti-inflammatory effect	Not indicated
Sargiacomo et al. [52]	Doxycycline, azithromycine	Research perspective	Protein synthesis, viral replication inhibition	Not indicated
Bonzano et al. [92]	Doxycycline	Opinion	Protein synthesis, viral replication inhibition, and immunomodulatory effect	Not indicated
Karampela and Dalamaga [64]	Levofloxacin, moxifloxacin	Opinion	Immunomodulatory effect	Not indicated
Marciniec et al. [65]	Ciprofloxacin, moxifloxacin	In silico study	Viral replication inhibition	Not indicated
Durojaiye et al. [81]	Cefuroxime	In silico study	Interaction between viral spike protein and ACE2 receptors and viral replication inhibition	Not indicated
Chalichem et al. [73]	Aminoglycosides		Membrane fusion inhibition	Not indicated

### Macrolides (azithromycin and clarithromycin)

Macrolides are a class of broad-spectrum antibiotics of large molecular size, including among others erythromycin, clarithromycin, and azithromycin [19]. Macrolides have generally a good tolerability profile [19]. Drugs in this class are used primarily to treat both local and systemic infections, including infections of the skin, eyes, respiratory tract, gastrointestinal tract, and genital tract [19]. In addition to their antibacterial activities, numerous macrolides antibiotics have been shown to possess considerable antiviral activities [20–24].

Among the antibiotics used against COVID-19, azithromycin is the most frequently used. Azithromycin is a broad-spectrum, macrolide antibiotic [25]. It has aa long half-life and excellent tissue penetration [25]. Numerous studies have previously reported the antiviral activity of azithromycin against Ebola virus and Zika virus [20–22]. In the management of COVID-19, azithromycin is used alone or in combination with hydroxychloroquine [26–29]. It is recommended for use at the early stage of the disease especially before the on-set of complications [30, 31]. Studies have however shown that the efficacy of azithromycin alone or in combination with hydroxychloroquine in COVID-19 remain highly controversial and not widely accepted [28, 32].

The mechanism through which azithromycin exerts its antiviral activity is still unknown. Nevertheless, numerous mechanisms have been proposed. It has been proposed that azithromycin may inhibit acidification of endosome during viral replication and infection (Table 1) [33]. As a weak base, azithromycin accumulates in endosomal vesicles, increasing the pH level. Endosomal acidification and cleavage processes are required for the viral replication and infection. Another possible target for azithromycin is the un-coating step during viral infection [34]. This step in the virus life cycle also requires acidic environment. Furthermore, based on their anti-inflammatory and immunomodulatory effects, azithromycin has been proposed as option for patients with virus infections and inflammatory basis [34]. Azithromycin reduces the production of pro-inflammatory cytokines such as interleukins-8 (IL-8), IL-6, tumor necrotic factor alpha (TNF-α), matrix metalloproteinases (MMPs) [35]. It also reduces oxidative stress, and modulate T-helper functions [35].

Because of the comparable mode of action of azithromycin and clarithromycin, clarithromycin was the second macrolide antibiotic proposed for the treatment of COVID-19 patients [23, 24]. However, subtle differences exist in the pharmacodynamics, pharmacokinetics, drug interaction, and safety of the two drugs [36]. Studies have demonstrated the antiviral properties of clarithromycin in seasonal influenza virus infection [23, 24]. A recent study has shown that clarithromycin in combination with chloroquine significantly improved clinical condition of a patient with SARS-coronavirus-2 infections and the patient tested negative by rRT-PCR test in less than 14 days [37].

Similar to azithromycin, the exact antiviral mechanism of clarithromycin has also not been determined. It has however been suggested that clarithromycin “suppresses infection-related inflammation and reduces vascular hyper-permeability by suppressing the induction of monocyte chemoattractant protein-1 (MCP-1) and matrix metalloproteinases-9 (MMP-9)” [24].

### Glycopeptide (teicoplanin)

Glycopeptides are a group of large molecular weight antibiotics that inhibit transglycosylation and transpeptidation, the later stage of bacterial cell-wall peptidoglycan biosynthesis [38]. This class includes vancomycin and teicoplanin [38]. They are the last-line antibiotic for treatment of severe infections caused by multidrug resistant Gram-positive pathogens, particularly methicillin‐resistant *Staphylococcus aureus* (MRSA) and Enterococci. In addition to their antibacterial properties, glycopeptides and specifically teicoplanin have been shown to exhibit significant antiviral activities [39]. Previously, the antiviral activity of teicoplanin against Ebola virus, SARS-CoV, and MERS-CoV has been established. This has been suggested to be due to inhibition of entry of the viral particles into the cells [39, 40].

The potential activity of teicoplanin against SARS-CoV-2 was first postulated by Baron et al. [41]*.* In another study, teicoplanin at a dose of 6 mg/kg every 24 h for 10 days was found to be effective and safe for the treatment of 2019-nCoV virus infection [42].

The precise anti-viral mechanism of teicoplanin has also not been determined. It has however been suggested that teicoplanin potently block the entry of SARS-CoV-2 through the inhibition of the enzymatic activity of cathepsin L [43]. Based on this, the authors recommended the use of teicoplanin in both prophylaxis and therapeutic management of patients with SARS-CoV-2 infection [43].

### Tetracyclines (doxycycline, eravacycline)

Tetracyclines are broad spectrum bacteriostatic and lipophilic antibiotics with high tissue penetration in the lungs [44]. These drugs exerts their activity by binding to bacterial ribosomes and interact with conserved region of bacterial 16S ribosomal RNA (rRNA) leading to inhibition of bacterial protein synthesis, by preventing the association of aminoacyl-tRNA with the bacterial ribosome [44]. Tetracyclines antibiotics have high activity against Gram-positive and -negative bacteria, spirochetes, obligate intracellular bacteria, as well as protozoan parasites [44]. In addition to this, tetracyclines have a number of non‐antibiotic effects including substantial antiviral activities [45–47].

The antiviral activity of doxycycline was first described by Sturtz [47]. This has been further confirmed by other researchers [45, 46, 48, 49]. The antiviral effects of doxycycline may be due to up-regulation of zinc finger antiviral protein (ZAP), preventing the accumulation of viral RNA in the cytoplasm [50, 51]. Doxycycline as a senolytic drug could inhibit protein synthesis, senescence-associated secretory phenotype, viral replication, and prevent lung fibrosis [52]. Doxycycline may also exert anti-inflammatory effect in patients with viral infection by inhibiting pro-inflammatory cytokines, including IL-6 and tumor necrosis factor (TNF)-α [53]. The commonest morbid complication of SARS-CoV2- induced pneumonia are the hyper-inflammation and cytokine storm [54, 55]. Moreover, a computational model revealed that doxycycline is a potential drug candidate for SARS-CoV-2, by inhibiting the SARS-CoV-2 main proteinase (Mpro), also known as 3-chymotrypsin like protease (3CLpro) [56]. This 3CLpro plays important roles in proteolytic processing of viral polyproteins, essentially in the replication of RNA viruses, including SARS coronavirus [57]. In another computational study, eravacycline, a synthetic halogenated tetracycline class antibiotic was found as the “second-best repurposed drug candidate” for SARS-CoV-2 main protease [58].

### Fluoroquinolones (ciprofloxacin, moxifloxacin and levofloxacin)

Fluoroquinolones are a class of broad-spectrum synthetic antibiotics. Fluoroquinolones inhibited the activities of prokaryotic DNA gyrase—topoisomerase II and topoisomerase IV, which are essential for DNA replication and transcription [59]. This class of antibiotics has high activity against Gram-negative and Gram-positive bacteria, mycobacteria, and anaerobes bacteria [59]. In addition to their antibacterial effects, the potential antiviral property of fluoroquinolones against both DNA and RNA viruses is also well documented [60–63].

Studies have demonstrated the potential action of fluoroquinolones for the treatment of SARS-CoV-2 associated pneumonia and called for randomized clinical trials of respiratory fluoroquinolones such as ciprofloxacin, moxifloxacin and levofloxacin [64, 65]. Interestingly, these drugs were also recommended in the treatment of community-acquired pneumonia in COVID-19 patients [66].

As a chemical derivative of quinoline, the prodrome of chloroquine, the antimalarial drug which has been proven effective in COVID-19 patients [12, 26], fluoroquinolones may exert antiviral activity in the treatment of SARS-CoV-2 infection. Ciprofloxacin and moxifloxacin may bind to SARS-CoV-2 3CLpro which is involved in the inhibition of SARS-CoV-2 replication [65]. Furthermore, fluoroquinolones also have immune-modulatory activity leading to attenuation of cytokines response, essential for the infamous cytokines storm syndrome [67, 68].

### Aminoglycosides

Aminoglycosides are one of the oldest classes of antibiotics. Aminoglycosides exert antibacterial activity by binding specifically to the aminoacyl site of 16S ribosomal RNA (rRNA) within the 30S ribosomal subunit and interfere with protein synthesis [69]. Aminoglycosides have relatively high frequency of nephrotoxicity and ototoxicity [70]. Gentamycin, tobramycin, and amikacin are the most prescribed aminoglycosides in clinical practice [70]. These bactericidal antibiotics have high activity against Gram-positive and Gram-negative bacteria and mycobacteria [70]. Additionally, aminoglycosides have a number of proven non-antibacterial therapeutic uses including antiviral properties [71, 72].

According to Chalichem et al., the effectiveness of aminoglycosides against SARS-CoV-2 may be due to production of retrocyclins, a functional peptide produced from human theta defensins, which inhibits cellular fusion and aggregation of SARS-CoV-2 [73]. Humans defensins exert a well-documented antiviral activity against both enveloped and non-enveloped viruses [74–78].

Unfortunately, the adverse impact of SARS-CoV-2 infection on olfaction [79] counteract with the well-known ototoxicity associated with the use of aminoglycosides. Consequently, the clinical use of aminoglycosides in the management of patients with SARS-CoV-2 infection was discouraged [79].

### Cephalosporins (cefuroxime)

Cephalosporins in combination with beta-lactamase inhibitors are commonly used in elderly patients with community-acquired pneumonia [80]. Cefuroxime is a second-generation cephalosporin antibiotic with broad spectrum activity. It generally has good tolerability and safety profiles and it is used to treat respiratory and genitourinary tract infections, and Lyme disease. In a recent review, the authors have shown in-silico evidence of the potential action of cefuroxime against three SARS-CoV-2 proteins, including main protease, RNA-dependent RNA polymerase, and angiotensin-converting enzyme 2 (ACE2)-Spike complex [81]. However, no in-vitro or human clinical trial has been conducted to establish the proprieties of this finding.

## Conclusion

Antibiotic repurposing is one of the therapeutic strategies being employed in the clinical management of COVID-19. This is aimed at either the resolution of any bacterial infections co-existing with the COVID-19 infections or exploitation of its potential antiviral properties. Though some of these antibiotics have shown promising results, their use remains highly controversial and not widely accepted. Moreover, the precise antiviral mechanism of most of these antibiotics has not yet been determined. Considering the positive association between heavy antibiotic use and worsening of antibiotic resistance crisis, efforts should be made to strengthen antibiotic stewardship at both national and sub-national levels so as to reduce the long and short impact of antibiotic use in COVID-19 on the antibiotic resistance crisis. Also, data are needed to increase the body of evidence and the clinicians’ confidence in the use of antibiotics for COVID-19 diseases.

## Data Availability

All relevant data are available within the paper. https://doi.org/10.6084/m9.figshare.12690077.v1.
